# Analgesic Effect of Rectus Sheath Block Versus Local Infiltration Analgesia in Laparoscopic Sleeve Gastrectomy: A Randomized Controlled Trial

**DOI:** 10.1007/s11695-025-08405-3

**Published:** 2025-12-04

**Authors:** Artid Samerchua, Kanokkan Tepmalai, Bandhuphat Chakrabandhu, Kittitorn Supphapipat, Panuwat Lapisatepun, Prangmalee Leurcharusmee, Kullaphun Prapussarakul, Thidarut Jinadech, Kotchakorn Jungsakulrujirek, Mullika Wanvoharn

**Affiliations:** 1 Department of Anesthesiology, Faculty of Medicine, Chiang Mai University, Chiang Mai, Thailand; 2 Department of Surgery, Faculty of Medicine, Chiang Mai University, Chiang Mai, Thailand

**Keywords:** Sleeve gastrectomy, Bariatric surgery, Laparoscopy, Nerve block, Analgesia, Obesity

## Abstract

**Background:**

Rectus sheath block (RSB) provides reliable anesthesia to the anteromedial abdominal wall and may offer effective pain control following laparoscopic sleeve gastrectomy (LSG). This study aimed to compare the efficacy of RSB versus local infiltration analgesia (LIA), hypothesizing that RSB would provide superior pain relief.

**Methods:**

In this randomized controlled trial, patients with obesity undergoing LSG received either bilateral ultrasound-guided RSB performed by an anesthesiologist or LIA administered by a surgeon, following anesthesia induction. The primary outcome was intraoperative fentanyl consumption. Secondary outcomes included postoperative pain scores [Numeric Rating Scale (NRS), 0–10], cumulative morphine consumption, and recovery metrics over 48 h.

**Results:**

Sixty-nine patients were analyzed (RSB: 35; LIA: 34). Intraoperative fentanyl use was lower with RSB (median difference: − 25 mcg; 95% CI: − 50 to 0; *p* = 0.008). RSB reduced pain scores at rest at 0 h (–2 points; *p* = 0.001), 1 h (–1 point; *p* = 0.009), and 12 h (–1 point; *p* = 0.022), and pain during movement at 0 h (–3 points; *p* < 0.001), 12 h (–1 point; *p* = 0.043), and 36 h (–1 point; *p* = 0.019). Pain scores were otherwise comparable. Fewer RSB patients reported moderate-to-severe pain at 0 h and 1 h (rest: *p* = 0.006 and 0.019; movement: *p* < 0.001 and 0.033). Postoperative morphine use and recovery metrics were similar between groups.

**Conclusion:**

Pre-incisional RSB demonstrated an intraoperative opioid-sparing effect and superior early postoperative analgesia compared with LIA, supporting its role as a component of multimodal analgesia for LSG.

## Key Points

RSB provides reliable analgesia for the upper anteromedial abdomen.

RSB did not reduce opioid use after laparoscopic sleeve gastrectomy.

RSB can reduce early postoperative pain following sleeve gastrectomy.

## Introduction

Laparoscopic sleeve gastrectomy (LSG) is a widely performed bariatric metabolic surgery (BMS) that offers sustained weight loss, improved metabolic outcomes, and increased life expectancy [1]. Despite its minimally invasive approach, LSG is often associated with moderate-to-severe postoperative pain, frequently necessitating substantial opioid use [2, 3]. This increases the risk of opioid-induced ventilatory depression and postoperative nausea and vomiting (PONV), potentially delaying recovery [4, 5].

To minimize perioperative opioid use, the Enhanced Recovery After Surgery (ERAS^®^) Society recommends opioid-sparing strategies through multimodal analgesia, including the use of regional anesthesia (RA) and local anesthetic (LA) techniques [1]. Although methods such as local infiltration analgesia (LIA), transversus abdominis plane block (TAPB), and erector spinae plane block (ESPB) have been explored, the evidence remains inconclusive, and technical challenges persist in patients with obesity [6–9].

Rectus sheath block (RSB) provides direct blockade of the anterior branches of the thoracoabdominal nerves [8, 10]. Accordingly, RSB is well-suited for analgesia of the anteromedial abdominal wall, including regions commonly affected by laparoscopic upper abdominal surgery [11–13]. Despite these anatomical advantages, clinical evidence supporting the use of RSB specifically for LSG remains limited [8]. Therefore, this randomized controlled trial aimed to compare the analgesic efficacy of RSB versus LIA. We hypothesized that pre-incisional RSB would provide superior analgesia. The primary outcome was intraoperative opioid consumption, while secondary outcomes included postoperative pain intensity, opioid use, and recovery parameters.

## Methods

This study was designed as a prospective, randomized, controlled superiority trial comparing pre-incisional RSB with LIA in patients undergoing LSG between August 2024 and July 2025. The study was approved by the local ethics committee and registered at ThaiClinicalTrials.gov (TCTR20241026004) on 1 October 2024 (submitted by Panuwat Lapisetpan). Informed consent was obtained from all individual participants included in the study. This manuscript adheres to the applicable CONSORT guidelines.

Inclusion criteria were patients scheduled to undergo LSG, aged 20 to 65 years, with a BMI > 35 kg/m². Exclusion criteria included known allergies to any study drugs (e.g., local anesthetics, opioids, acetaminophen); recent or chronic opioid use; underlying chronic kidney disease, liver cirrhosis, major cardiac disease, pre-existing neurological deficits, or a documented diagnosis of chronic pain syndrome (e.g., complex regional pain fibromyalgia).

All patients were fasted per standard care. Antihypertensive agents on the day of surgery were managed according to standard institutional recommendations (e.g., contrinuation of most agents, withholding of ACE inhibitors/ARBs or diuretics when clinically indicated). Upon arrival in the operating room, an attending anesthesiologist, blinded to the patient’s group assignment, supervised anesthesia induction. All patients received general anesthesia (GA) with propofol 2–3 mg/kg, fentanyl 1 mcg/kg, and succinylcholine 1 mg/kg, followed by tracheal intubation. GA was maintained with sevoflurane or desflurane to achieve a MAC of 0.7-1.0, and all patients received rocuronium for muscle relaxation. Once the patient’s condition stabilized, the attending anesthesiologist exited the operating room. A regional anesthesiologist and their team then assumed care. The group assignment was revealed by opening a sealed envelope opened by the regional anesthesiologist.

### Intervention

**Fig. 1:**
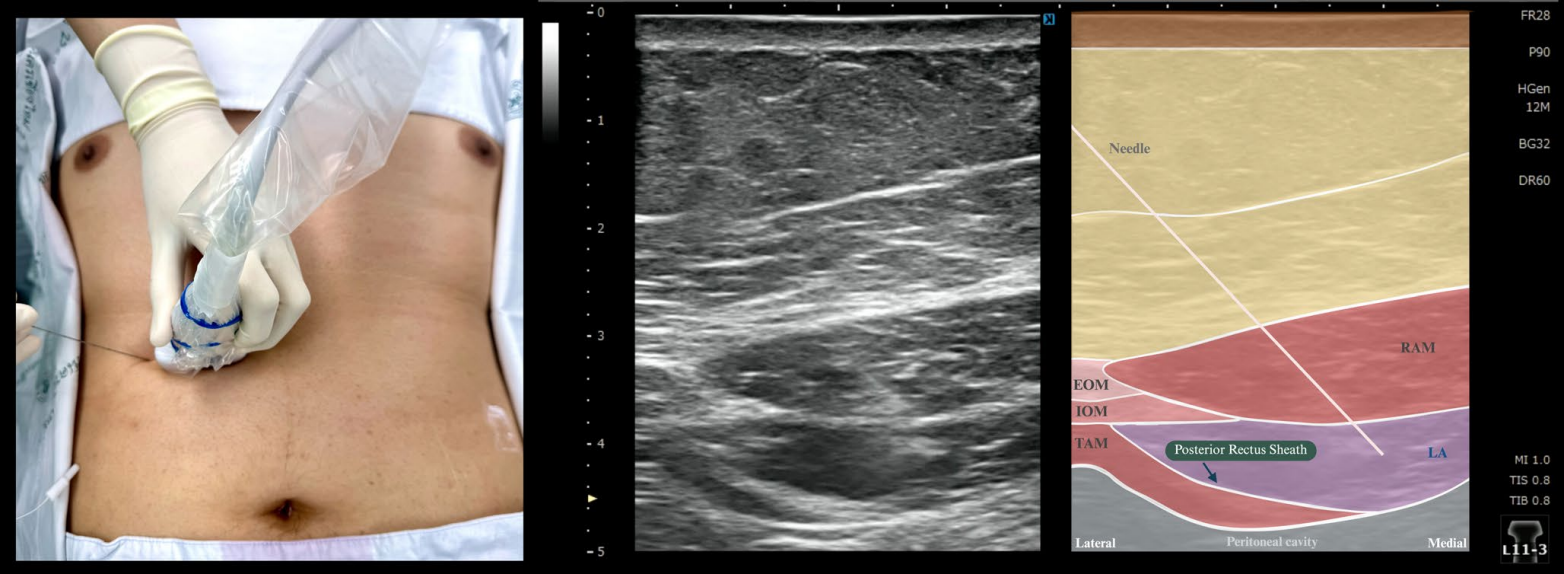
Performance of RSB. *RAM* rectus abdominis muscle; *EOM* external oblique muscle; *IOM* internal oblique muscle; *TAM* transversus abdominis muscle; *LA* Local Anesthetic

Pre-incisional RSB was performed under sterile conditions by a regional anesthesiologist. Under ultrasound guidance, the transducer was placed transversely over the upper third of the distance between the epigastrium and umbilicus to visualize the lateral edge of the rectus abdominis muscle (RAM). A 22-gauge, 100-mm block needle (Stimuplex A100, B. Braun Medical AG, Melsungen, Germany) was inserted in-plane from lateral to medial, targeting the interfascial plane between the RAM and its posterior sheath. Correct needle placement was confirmed by injecting normal saline, producing visible separation of the RAM from its posterior sheath (Fig. 1). Subsequently, 20 mL of an LA mixture (0.25% bupivacaine, 1% lidocaine with epinephrine (10 mcg/mL), and dexamethasone (10 mg)) was slowly injected. The procedure was performed bilaterally (total LA volume 40 mL). Patients were then prepped and draped for surgery.

In the LIA group, after surgical preparation and draping, the same LA mixture and total volume (40 mL) were administered by the surgeons at the port sites, trocar sites, and within the preperitoneal plane under direct visualization using a laparoscopic camera. Specifically, 15 mL was injected at the 20-mm trocar port, 10 mL at the camera port site, 5 mL at the liver retraction port, and 5 mL at each of the remaining ports.

The regional anesthesiologist managed the patient during the RSB or LIA and was not permitted to administer fentanyl. After completion of the procedure and once all surgical instruments were prepared, the attending anesthesiologist who was blinded to group allocation resumed intraoperative care and managed anesthesia for the LSG.

Intraoperatively, intravenous fentanyl (25–50 mcg) was administered as needed when heart rate or systolic blood pressure increased by more than 20% from baseline. Adequacy of analgesia was further assessed clinically, with pupillary constriction used as an additional indicator. Hemodynamic changes such as tachycardia due to pneumoperitoneum, hypovolemia from the reverse Trendelenburg position, or inadequate anesthetic depth were managed with appropriate interventions, including increased minute ventilation, fluid resuscitation, administration of appropriate antihypertensive agents e.g., esmolol or cardipine, or adjustment of volatile concentration.

Postoperatively, all patients received care according to the ERAS guideline [1], including acetaminophen 1000 mg IV and ondansetron 8 mg IV every six hours for four scheduled doses, followed by as-needed dosing for pain and PONV. Postoperative pain was assesed using the numeric rating scale (NRS; 0 = no pain, 10 = worst imaginable pain) by trained nurses who were blinded to group allocation. Morphine (0.03 mg/kg IV) was administered in the post-anesthesia care unit (PACU) every 15 min as needed for an NRS pain score >4, both at rest and during movement. Pain intensity was reassessed after each dose at 15-minute intervals to ensure adequate analgesia. If patients reported an NRS score of 7 or higher, an additional rescue bolus of morphine was administered immedately, and reassessment was performed within 15 min to prevent prolonged severe pain. After discharge from the PACU, morphine (0.03 mg/kg IV) was avaliable on the ward every four hours as needed, with earlier rescue permitted for breakthrough pain.

### Outcome Measurements

All outcomes were evaluated by a blinded assessor. The primary outcome was intraoperative fentanyl consumption. Secondary outcomes included postoperative pain scores recorded at 0 h (immediately in the PACU) and 1, 6, 12, 24, 36, and 48 h. Pain intensity was categorized as no-to-mild (NRS 0–3), moderate (NRS 4–6), and severe (NRS 7–10). Cumulative morphine consumption over 48 h was also recorded. Recovery parameters included time to ambulation, time to first oral intake, and incidence of PONV. Sensory block assessment was performed in the PACU by a blinded assessor using a cold sensation test with an ice pack, evaluating changes in cutaneous cold perception over the anteromedial abdominal wall, after patients had regained full consciousness and achieved optimal pain control.

Block-related complications, such as bleeding, hematoma, intra-abdominal puncture, or local anesthetic systemic toxicity, were documented by a non-blinded operator.

### Statistical Method

Data were analyzed using STATA version 16 (StataCorp, 2019). Normality was assessed with the Shapiro–Wilk test. Continuous variables are presented as mean ± SD with 95% confidence intervals (CI) or median (Q1-Q3), and categorical variables as counts and percentages. Fisher’s exact test, Student’s t-test, or Mann–Whitney U test were used as appropriate. Median differences with 95% CI were estimated using the Hodges–Lehmann method. Postoperative NRS scores were analyzed using two-way repeated measures ANOVA. Analyses followed an intention-to-treat approach, with *p* < 0.05 considered statistically significant.

The sample size calculation was based on data from a pilot study of 20 patients (10 in each group), which demonstrated mean ± SD intraoperative fentanyl consumption of 110 ± 50 mcg in the RSB group and 150 ± 58.6 mcg in the LIA group. With α = 0.05 and β = 0.2, 30 patients per group were required to detect a significant difference. To account for a potential 20% dropout, 35 patients were enrolled in each group.

## Results

**Fig. 2:**
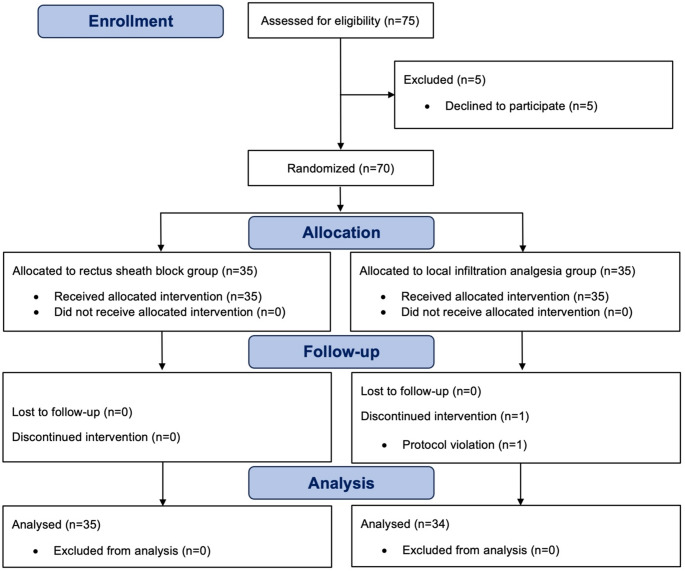
Study flow diagram

During the study period, 75 patients were screened for eligibility. Five patients were excluded after declining to participate. The remaining 70 patients were randomized to receive either RSB (*n* = 35) or LIA (*n* = 35) (Fig. 2). All patients received the assigned intervention; however, one patient in the LIA group was excluded from analysis due to an incorrect intraoperative fentanyl dose. Thus, 69 patients were included in the per-protocol analysis, which included all randomized participants who received the allocated intervention and were eligible for outcome assessment. Baseline patient characteristics were comparable between groups, as shown in Table 1. None of the patients was taking beta-blocking agents. Anesthesia and operative times were comparable between groups, with no significant difference observed.

**Table 1:** Patient characteristics

	RSB Group (*n* = 35)	LIA Group (*n* = 34)	*p*-value
Age (years)	32 (30–37)	34.5 (28–44)	0.174^†^
Men	25 (71.4)	23 (67.7)	0.733^‡^
BMI (kg/m^2)^	42.9 ± 6.0	43.4 ± 8.6	0.810^§^
Associated medical problems			
Hypertension	17 (48.6)	19 (55.9)	0.543^‡^
Diabetic mellitus	16 (45.7)	13 (38.2)	0.529^‡^
Dyslipidemia	16 (45.7)	19 (55.9)	0.398^‡^
OSA	32 (91.4)	29 (85.3)	0.426^‡^
Duration of surgery (min)	90 (80–120)	120 (75–135)	0.287^†^
Duration of anesthesia (min)	135 (120–170)	162.5 (120–180)	0.301^†^

Data are presented as numbers (%), median (Q1-Q3), or mean ± SD

*P*-values correspond to ^†^Mann-Whitney U test, ^‡^ Fisher’s exact test, and ^§^ Student’s *t*-test

*BMI* body mass index; *ASA* American Society of Anesthesiologists; *OSA* obstructive sleep apnea; *RSB* rectus sheath block; *LIA* local infiltration analgesia

**Fig. 3:**
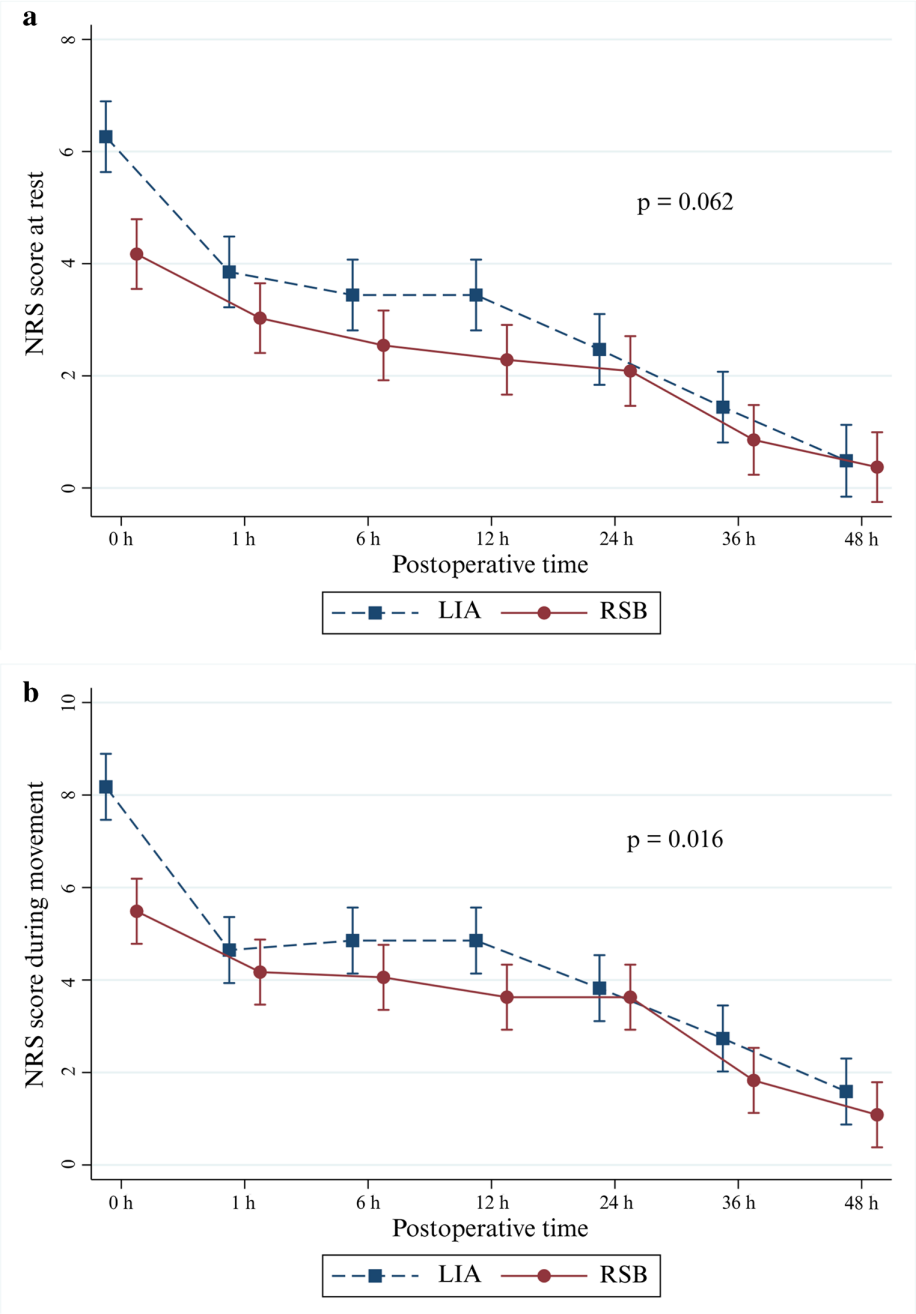
Adjusted predicted postoperative pain scores over time with 95% CI for the RSB and LIA Groups at Rest (**a**) and During Movement (**b**)

**Fig. 4:**
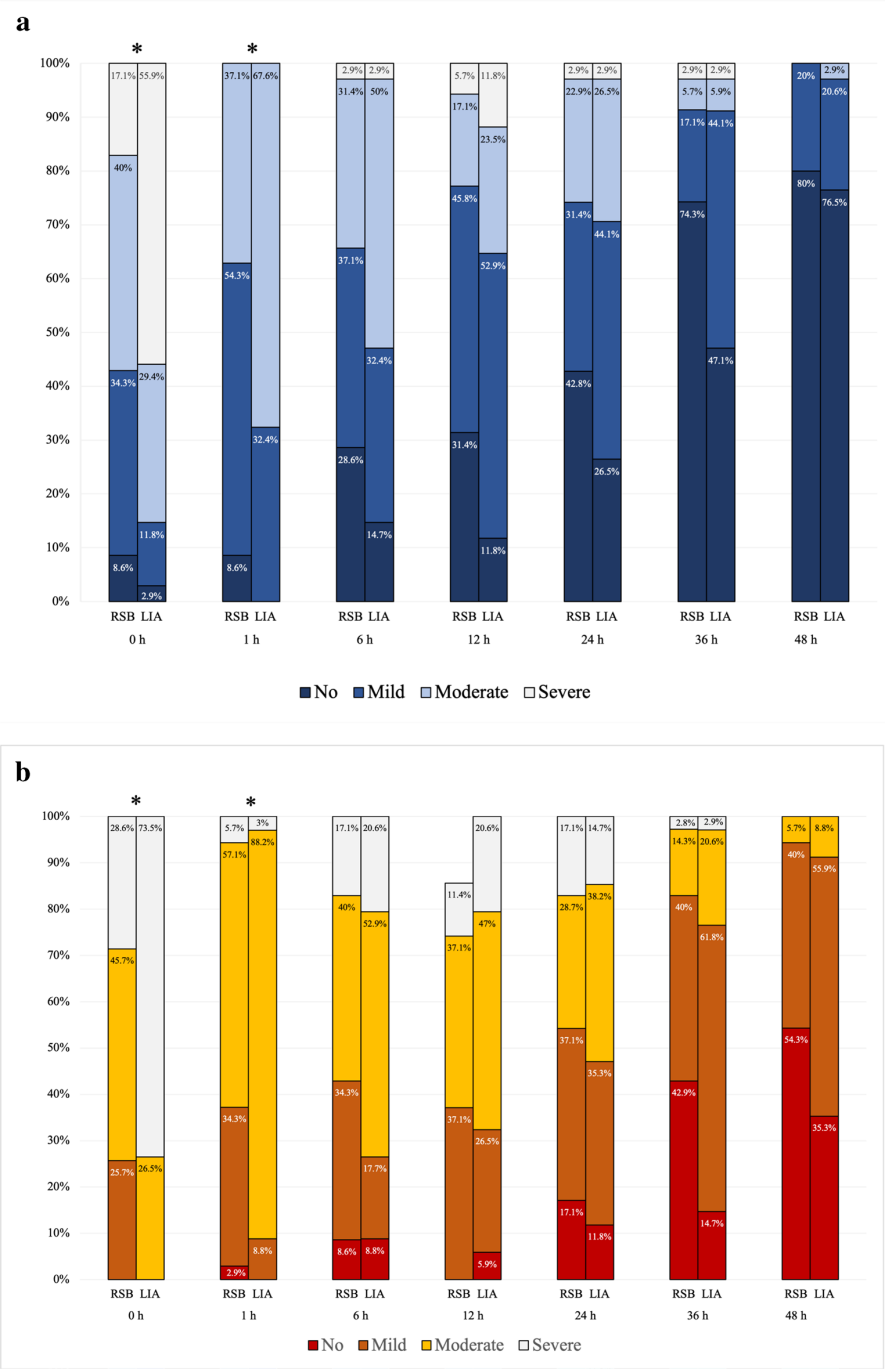
Proportion of pain severity in the RSB and LIA groups across postoperative time points at rest (**a**) and during movement (**b**). *Indicates a statistically significant difference between groups

For the primary outcome, intraoperative fentanyl consumption was significantly lower in the RSB group compared to the LIA group, with a median difference of − 25 mcg (95% CI: − 50 to 0; *p* = 0.008) (Table 2). Regarding secondary outcomes, cumulative postoperative morphine consumption was comparable between groups. Postoperative NRS pain scores are shown in Table 3. Significantly lower pain scores were observed in the RSB group at rest at 0, 1, and 12 h, and during movement at 0, 12, and 36 h. Pain during movement also differed significantly between groups over time (*p* = 0.016), favoring the RSB group (Fig. 3). Stacked bar analyses (Fig. 4) further demonstrated the analgesic advantage of RSB, with a significantly lower proportion of patients in the RSB group reporting moderate-to-severe pain at 0 h and 1 h, both at rest (*p* = 0.006 and 0.019) and during movement (*p* < 0.001 and 0.033).

**Table 2:** Perioperative outcomes

		RSB Group (*n* = 35)	LIA Group (*n* = 34)	Median Difference (95% CI)	*p*-value
Intraoperative fentanyl consumption (mcg)		125 (100–150)	150 (125–200)	-25 (-50 to 0)	0.008
Cumulative postoperative morphine consumption (mg)					
In PACU		4 (3–6)	6 (3–8)	-1 (-3 to 0)	0.093
At 24 h		6 (3–9)	8 (4–9)	0 (-3 to 1)	0.379
At 48 h		6 (3–12)	8 (6–9)	-1 (-3 to 0)	0.172
Time to off-bed activity (h)		19 (15–22)	20 (16–22)	0 (-3 to 1)	0.514
Time to first oral feeding (h)		20 (17–24)	20 (18–22)	0 (-2 to 2)	0.885
Time to first flatus (h)		21 (20–23)	22.5 (20–24)	-1 (-3 to 0)	0.107

Data are presented as median (Q1-Q3)

The median difference was calculated as RSB minus LIA using the Hodges–Lehmann estimator

*P-*values correspond to the Mann–Whitney U test

*PACU* Post Anesthesia Care Unit; *RSB* rectus sheath block; *LIA* local infiltration analgesia

**Table 3:** Postoperative pain (NRS) scores

	RSB Group (*n* = 35)	LIA Group (*n* = 34)	Median Difference (95% CI)	*p*-value
Postoperative pain (NRS 0–10) at rest				
0 h (at PACU)	4 (2–6)	7 (4–8)	-2 (-3 to -1)	0.001
1 h	3 (2–4)	4 (3–4)	-1 (-1 to 0)	0.009
6 h	3 (0–4)	4 (3–5)	-1 (-2 to 0)	0.074
12 h	2 (0–3)	3 (2–5)	-1 (-2 to 0)	0.022
24 h	2 (0–4)	2 (0–4)	0 (-2 to 0)	0.421
36 h	0 (0–1)	1 (0–2)	0 (-2 to 0)	0.050
48 h	0 (0–0)	0 (0–0)	0 (0 to 0)	0.720
Postoperative pain (NRS 0–10) during movement				
0 h (at PACU)	5 (3–8)	10 (6–10)	-3 (-4 to -2)	< 0.001
1 h	4 (3–5)	5 (4–5)	-1 (-1 to 0)	0.111
6 h	4 (2–6)	5 (3–6)	-1 (-2 to 0)	0.144
12 h	3 (2–5)	4 (3–6)	-1 (-2 to 0)	0.043
24 h	3 (2–5)	4 (2–5)	0 (-2 to 1)	0.636
36 h	2 (0–3)	3 (2–3)	-1 (-2 to 0)	0.019
48 h	0 (0–2)	2 (0–2)	0 (-1 to 0)	0.141

Data are presented as median (Q1-Q3)

The median difference was calculated as RSB minus LIA using the Hodges–Lehmann estimator

*P-*values correspond to the Mann–Whitney U test

*NRS* Numeric Rating Scale; *PACU* Post Anesthesia Care Unit; *RSB* rectus sheath block; *LIA* local infiltration analgesia

**Fig. 5:**
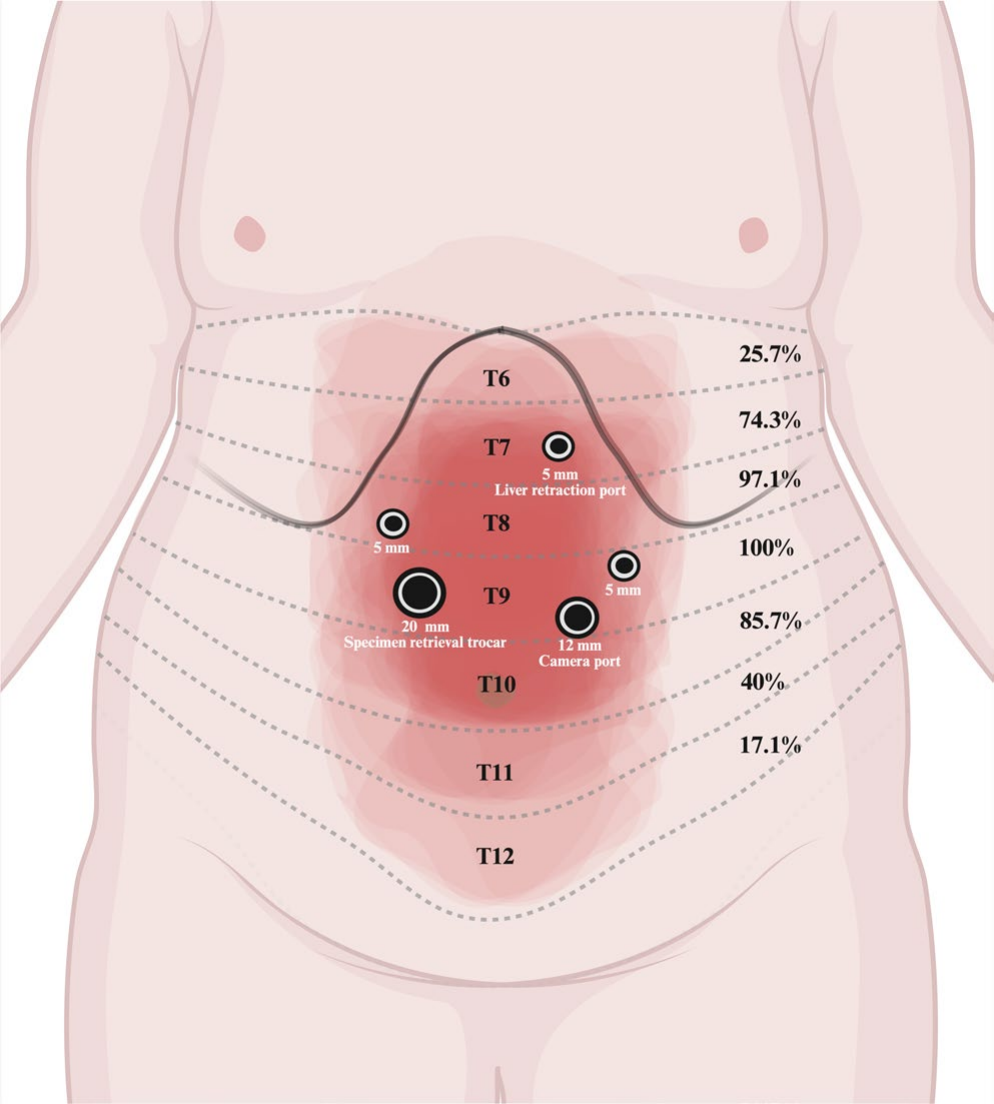
Percentage of dermatomal sensory block achieved with RSB relative to the surgical incision (created in BioRender.com)

A schematic illustration of the percentage of dermatomal sensory block achieved with RSB relative to surgical incision sites is shown in Fig. 5. Bilateral sensory blockade from RSB consistently covered the T8–T10 dermatomes in over 85% of patients. Notably, none of the patients in the LIA group exhibited dermatomal cutaneous sensory loss on dermatomal testing.

Recovery outcomes were comparable between the two groups (Table 2**)**. Furthermore, the incidence of PONV did not differ significantly between groups (71.4% in RSB vs. 64.7% in LIA; *p* = 0.549). No complications related to either RSB or LIA were reported.

## Discussion

To our knowledge, this is the first study to compare the effects of pre-incisional RSB and LIA in patients undergoing LSG. The primary outcome, intraoperative fentanyl consumption, was significantly lower in the RSB group than in the LIA group. This reduction may be attributed to the broader somatic coverage achieved by RSB, which anesthetizes the abdominal wall, including the RAM and parietal peritoneum, both of which are susceptible to shearing injury and contribute to postoperative pain [8, 11, 13]. In contrast, the effectiveness of LIA may have been limited in patients with obesity due to incomplete distribution of LA, likely influenced by thick subcutaneous tissue [14, 15]. Although the reduction in fentanyl use was statistically significant (an absolute difference of 25 mcg, representing a 17% reduction), it did not meet the threshold for the minimal clinically important difference (a 30% reduction in opioid use) [16]. This modest benefit may reflect the limited effect of RSB on visceral pain, particularly that arising from gastrectomy, peritoneal stretching, or pneumoperitoneum during laparoscopy [17, 18].

LIA has been associated with reduced postoperative opioid requirements and improved early mobilization in BMS [6]. However, its analgesic effect is often short-lived, and findings across studies remain inconsistent [6, 19]. In LSG, Aldohayan et al. reported that LIA reduced pain scores in the PACU and at 12 h postoperatively, with lower morphine consumption compared to placebo [14]. In contrast, our study found no significant difference in postoperative morphine use between groups, but LIA was less effective than RSB in reducing postoperative pain. The RSB group demonstrated both statistically and clinically meaningful reductions (a two-point difference) in NRS pain scores at rest and during movement at 0 h postoperatively [16]. This superior analgesia likely reflects RSB’s direct blockade of the RAM and overlying tissues, with additional benefit from its muscle relaxation effect [10, 17, 18]. These findings align with a recent meta-analysis showing that RSB significantly reduces pain scores at rest and during movement within the first 0–2 h after laparoscopic surgery [13].

Laparoscopic BMS is frequently associated with significant postoperative pain, with up to 80% of patients reporting moderate-to-severe pain in the PACU [2, 3]. Although LIA or RA can alleviate incisional pain, patients frequently report upper abdominal discomfort, likely attributed to visceral pain [3, 7, 9]. Mucosal edema and restricted gastric volume may further intensify pain during the first 24–48 h [20]. While NSAIDs are effective opioid-sparing agents. Concerns regarding marginal ulceration and anastomotic leaks led to their exclusion from our protocol [1, 3]. Inadequate early analgesia, particularly in the PACU, may worsen pain trajectories and increase later opioid use [2, 3]. Our findings support RSB over LIA, with fewer patients in the RSB group experiencing moderate-to-severe pain at 0–1 h postoperatively.

Optimal RA for intra-abdominal surgery should provide both somatic and visceral analgesia, as achieved with thoracic epidural or paravertebral blocks [10]. However, these techniques are technically challenging in patients with obesity and carry risks such as hypotension, neurological injury, and pneumothorax [15, 17]. Alternatively, the ESPB may offer both somatic and visceral analgesia via indirect paravertebral spread; however, Jinaworn et al. found no significant analgesic benefit of ESPB compared to no block [9].

As safer alternatives, TAPBs that target the T6–T9 intercostal plexus and the T9–L1 TAP plexus are commonly employed in BMS [10]. Although TAPB provides only somatic analgesia, it has been shown to reduce pain intensity and offer superior opioid-sparing effects compared to LIA within the first 24 h after BMS [7]. However, its analgesic efficacy remains inconsistent due to variations in injection site and technique, LA type and dose, and technical challenges in patients with obesity [7]. In this population, identifying the target fascial plane can be difficult due to excessive subcutaneous tissue, increasing the risk of inaccurate needle placement and block failure [21]. Salmonsen et al. recently reported that bilateral subcostal TAPB often produces heterogeneous, non-dermatomal sensory blockades with incomplete coverage of the upper anteromedial abdomen [22].

In contrast, the RSB which targets the rectus sheath plexus provided consistent bilateral T8–T10 sensory coverage in our study [10]. In patients with obesity, stretching of the RAM may limit the cranial spread of LA, and a volume of 20 mL may be insufficient to reliably anesthetize the T6–T7 dermatomes [23]. Further studies investigating higher injection volumes or modified techniques, such as double injections, may help achieve broader dermatomal coverage. Nevertheless, the sensory block area achieved with RSB in our study adequately covered most of the surgical incision sites used for LSG. Compared to TAPB, RSB is technically easier to perform, as it involves a more superficial needle path [10]. Moreover, RSB produces a lower peak plasma concentration of LA than TAPB, suggesting a potentially safer systemic profile [24]. Further comparative studies are warranted to evaluate the relative efficacy and safety of RSB, TAPB, and ESPB in BMS.

PONV is a common complication following LSG [1]. Patients are at high risk due to factors such as younger age, female sex, non-smoking status, impaired splanchnic perfusion during pneumoperitoneum, and surgically restricted gastric volume [25, 26]. The high incidence of PONV in both groups may also reflect the use of inhalation-based anesthesia, perioperative opioid administration, and possibly inadequate antiemetic prophylaxis in our protocol [25, 27]. Consequently, the potential advantage of RSB in reducing PONV could not be demonstrated in the present study.

This study has several limitations. First, intraoperative fentanyl consumption was used as a surrogate for analgesic efficacy; however, it have been influenced by confounding factors such as patient positioning, pneumoperitoneum, and hypercarbia. Although direct nociceptive monitoring would provide a more objective assessment, hemodynamic response and pupillary reflex dilation remain widely accepted surrogate indicators of intraoperative nociception, as used in this study [28–30]. Nevertheless, intraoperative fentanyl use, while commonly employed to estimate analgesic requirements, has inherent limitation as a primary outcome for evaluating the efficacy of preoperative RSB and LIA. It should therefore be interpreted cautiously and complemented by postoperative pain assessments to more comprehensively determine the block’s clinical benefit. Second, postoperative analgesia was managed according to a standardized morphine titration protocol. While this approach ensured consistency across groups, it may have been insufficient in some cases, as reflected by high NRS scores in the movement condition. This suggests that individualized escalation or patient-controlled analgesia (PCA) may be preferable in rountine practice. However, patients receiving LIA or RA for LSG typically require only a morphine loading dose in the PACU, followed by minimal to moderate use on the ward once pain is controlled, suggesting that PCA may not be essential in this context [2, 3, 6, 7, 9]. Third, we did not include a control group; therefore, the relative efficacy of RSB and LIA compared to no block could not be determined. Finally, as only patients undergoing LSG were included, the findings may not be generalizable to other BMS such as laparoscopic Roux-en-Y gastric bypass or proximal jejunal bypass.

## Conclusion

This trial demonstrated that pre-incisional RSB was associated with significantly lower early postoperative pain scores and fewer patients experiencing moderate-to-severe pain in the PACU compared with LIA. Although total postoperative opioid consumption and recovery profiles were comparable between groups, and the reduction in intraoperative fentanyl use did not meet the threshold for clinical significance, the consistent dermatomal coverage and favorable safety profile suggest that RSB may be a valuable component of multimodal analgesia protocols for LSG.

## Data Availability

Data furnished upon request.
